# Gastrointestinal complaints after Roux-en-Y gastric bypass surgery. Impact of microbiota and its metabolites

**DOI:** 10.1016/j.heliyon.2024.e39899

**Published:** 2024-10-31

**Authors:** Emma Custers, Yonta G.R. van der Burgh, Debby Vreeken, Frank Schuren, Tim J. van den Broek, Lars Verschuren, Ivo de Blaauw, Mark Bouwens, Robert Kleemann, Amanda J. Kiliaan, Eric J. Hazebroek

**Affiliations:** a Department of Bariatric Surgery, Vitalys, Rijnstate Hospital, Arnhem, the Netherlands; b Department of Medical Imaging, Anatomy, Radboud University Medical Center, Radboud Alzheimer Center, Donders Institute for Brain Cognition and Behaviour, Center for Medical Neuroscience, Nijmegen, the Netherlands; c Division of Human Nutrition and Health, Wageningen University & Research, Wageningen, the Netherlands; d Department of Metabolic Health Research, The Netherlands Organization for Applied Scientific Research (TNO), Leiden, the Netherlands; e Department of Surgery, Division of Paediatric Surgery, Radboudumc-Amalia Children's Hospital, Nijmegen, the Netherlands; f Dutch Digestive Foundation, Amersfoort, the Netherlands

**Keywords:** Obesity, Roux-en-Y gastric bypass, Gastrointestinal complaints, Microbiota, Metabolites

## Abstract

Unexplainable gastrointestinal complaints occasionally occur after Roux-en-Y Gastric Bypass (RYGB) surgery. We therefor investigated the impact of microbiota composition and metabolites on gastrointestinal complaints after RYGB. In the BARICO study (Bariatric surgery Rijnstate and Radboudumc neuroimaging and Cognition in Obesity), microbiota and metabolites were measured before surgery, and 6, and 24 months after surgery. Gastrointestinal complaints were assessed with the Irritable Bowel Syndrome Severity Scoring System (IBS-SSS) questionnaire 24 months after surgery. 65 participants (86.2 % female) with a mean age of 46.2 ± 6.0 years, and mean BMI of 41.2 ± 3.6 kg/m^2^ were included. According to the IBS-SSS questionnaire, 32.3 % had moderate/severe gastrointestinal complaints 24 months after surgery. Microbiota alpha diversity remained stable, while beta diversity significantly changed over time. Bile acids and short-chain fatty acids were significantly higher, and inflammatory markers significantly lower after surgery. *Barnesiella* sp., *Escherichia*/*Shigella* sp., and *Faecalibacterium prausnitzii* correlated positively, while *Akkermansia* sp correlated inversely with gastrointestinal complaints. Patients with mild and moderate/severe gastrointestinal complaints showed higher levels of GLC-3S. These findings suggest involvement of microbiota and metabolite changes in gastrointestinal complaints after surgery. However, it remains unclear whether bacteria influence gastrointestinal complaints directly or indirectly. Further exploration is required for development of interventions against gastrointestinal symptoms after surgery.

## Highlights

• Gastrointestinal complaints can occur after metabolic bariatric surgery (MBS).
• Microbiota beta diversity and metabolites change after MBS.
• Microbiome and metabolite changes may induce gastrointestinal complaints after MBS.

## Graphical abstract

**Figure:**
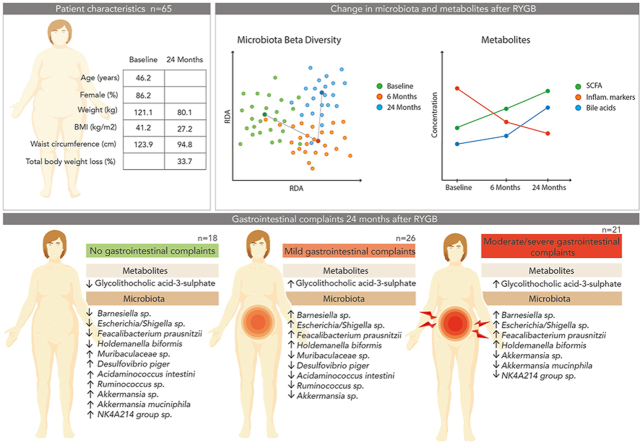

## 1 Introduction

Metabolic and bariatric surgery (MBS) is the most effective treatment for obesity, as it leads to prolonged weight loss and remission of comorbidities [1,2]. Roux-en-Y Gastric Bypass (RYGB) surgery is one of the most commonly performed bariatric procedures. However, up to 30 % of the patients suffers from recurrent gastrointestinal complaints after surgery. In some patients the cause of these gastrointestinal complaints remains unknown [3]. Moreover, it has been shown that the prevalence of gastrointestinal complaints, including chronic diarrhea, bloating and cramps [4] has more than doubled two years after RYGB [5,6], and often leads to a reduced quality of life [4].

Little is known about the underlying mechanisms responsible for gastrointestinal complaints after RYGB surgery, making it difficult to develop treatments. The gut, in particular the gut microbiota is thought to be responsible for gastrointestinal complaints [7,8]. RYGB changes the gut anatomy and thereby decreases intestinal transit time and absorptive capacity [7]. Moreover, anatomical rearrangements after RYGB induce a higher oxygen content in originally more distal parts of the gut and also change pH, creating a more aerobic environment [9]. Together with dietary changes after RYGB [10], these anatomical rearrangements may alter the gut microbiota composition, potentially leading to gastrointestinal complaints.

Patients with obesity often have dysbiotic microbiota which is thought to change after MBS [11,12]. After RYGB beneficial changes in the microbiota are reported, as *Akkermansia muciniphila*, bacteria that strengthen the intestinal barrier, and *Bacteroides,* anti-inflammatory bacteria, increased after surgery [9,13]. However, negative changes have also been reported as *Bifidobacterium*, bacteria known to digest fibers to and prevent infections, decreased [14], and *Fusobacteria*, bacteria associated with infections, increased after surgery [9]. Evidence for the role of gut microbiota on gastrointestinal complaints comes from studies regarding irritable bowel syndrome (IBS), in which a lower microbiota diversity is displayed in IBS patients [15,16]. Moreover, administration of probiotics alleviates gastrointestinal complaints after MBS [17].

The gut microbiota interacts with the host through production of small molecules, such as short-chain fatty acids (SCFA) and bile acids which may play a role in the pathogenesis of gastrointestinal diseases [18]. Bile acids exhibit amphipathic properties, important for lipid digestion and absorption in the small intestine [18]. Bile acids also act as signaling compounds that modulate inflammatory responses, stimulate visceral hypersensitivity, and increase intestinal motility [19]. SCFA are involved in several physiological functions including energy metabolism, regulation of the inflammatory response and maintenance of the mucosal integrity of the gut [20]. An altered microbiota can disturb the metabolite production and might stimulate the development of gastrointestinal diseases. Moreover, microbiota derived metabolites interact with nociceptors or pain-sensitive neurons with peripheral nerve terminals present in the intestinal wall [21], suggesting that they play an important role in visceral hypersensitivity and pain sensation.

To our knowledge, only one study investigated the effect of gut microbiota on gastrointestinal complaints after RYGB [4]. The possible effect of microbial metabolites however, has not been described previously. Therefore, this study aims to broaden the field by exploring the relation between microbiota composition as well as its metabolites, and the development of gastrointestinal complaints after RYGB in adults enrolled in the BARICO study (BAriatric surgery Rijnstate and Radboudumc neuroImaging and Cognition in Obesity) [22]. Identifying (bio)markers that predispose gastrointestinal complaints in RYGB patients might contribute to new therapeutic strategies to ameliorate and possibly prevent these complaints and ultimately, improve quality of life after MBS.

## 2 Material and methods

### 2.1 Description of study population

Within this observational study we analyzed data from participants of the BARICO study [22]. Between September 2018 and December 2020, 156 participants were recruited at Rijnstate Hospital (Arnhem, the Netherlands). Participants were 35–55 years old at recruitment and eligible for RYGB. All RYGB surgeries have been performed with an alimentary limb length of 100 cm, a biliopancreatic limb length of 150 cm and mesenteric defects were closed. Neurological or severe psychiatric illness, pregnancy, and treatment with antibiotics, probiotics or prebiotics 3 months before, or at any time point during the study were exclusion criteria.

Four weeks before surgery, and 6, and 24 months after surgery, participants were assessed via medical evaluation and blood and feces collection. To assess gastrointestinal complaints after RYGB, participants were asked to fill out the Irritable Bowel Syndrome Severity Scoring System (IBS-SSS) questionnaire 24 months after surgery. For this current study, subjects were only included if plasma and feces samples were collected at all timepoints, and the IBS-SSS questionnaire was completed.

**Fig. 1:**
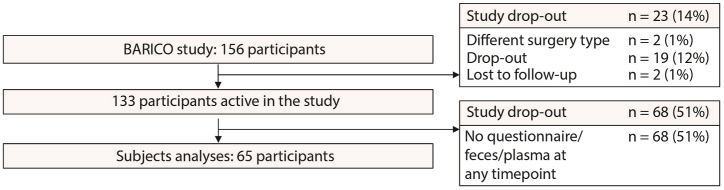
**Flowchart with included participants.** Of the 156 participants enrolled in the BARICO study, two participants were excluded due to a last-minute change of surgery (sleeve gastrectomy), nineteen participants dropped-out of the study before the 24 months follow-up and two participants were lost to follow-up. In total 133 participants finished their 24 months follow-up, of which 68 participants were excluded as they did not fill in the questionnaire or missed feces or blood samples at one of the timepoints.

Of the 156 participants, 2 participants were excluded due to a last-minute change of surgery, 19 participants dropped-out of the study due to various reasons and 2 participants were lost to follow-up. Of those who were still actively present in the study, 68 participants did not fill in the questionnaire, missed fecal samples or missed plasma samples, leaving 65 participants eligible for analysis (Fig. 1).

### 2.2 Standard protocol approvals, registration, and patient consents

The study was approved by the Medical Ethics Committee CMO region Arnhem - Nijmegen (NL63493.091.17) and by the local institutional ethics committee. The study was conducted in accordance with the Declaration of Helsinki ‘Ethical Principles for Medical Research Involving Human Subjects’ and in accordance with the guidelines for Good Clinical Practice (CPMP/ICH/135/95). All participants provided written informed consent. The study was prospectively registered in the Netherlands Trial Registry (NL7090: https://www.clinicaltrialregister.nl/nl/trial/28949). The Strengthening the Reporting of Observational Studies in Epidemiology (STROBE) reporting guideline was followed.

### 2.3 Medical examination

Anthropometric measurements included body weight, body mass index (BMI), waist circumference (WC), percentage total weight loss (%TWL) and blood pressure. BMI was calculated as weight in kg divided by height in meters squared. %TWL was defined as weight loss divided by initial weight. Blood pressure was measured in sitting position.

### 2.4 Biochemical analysis in plasma

Fasting blood samples were collected and stored at −80 °C 4 weeks before surgery, and 6 and 24 months after surgery. The analysis of bile acids (cholic acid (CA; μmol/l), chenodeoxycholic acid (CDC; μmol/l), deoxycholic acid (DCA; μmol/l), glycocholic acid (GCA; μmol/l), glycochenodeoxycholic acid (GCDC; μmol/l), glycodeoxycholic acid (GDC; μmol/l), glycolithocholic acid (GLC; μmol/l), glycolithocholic acid-3-sulphate (GLC-3S; μmol/l), taurocholic acid (TCA; μmol/l), tauro-chenodeoxycholic acid (TCDC; μmol/l), tauro-deoxycholic acid (TDC; μmol/l) and ursodeoxycholic acid (UDC; μmol/l)) and SCFAs (acetic acid, propionic acid, butyric acid, caproic acid, iso-butyric acid, methyl butyric acid, iso valeric acid, and valeric acid; μg/ml) was performed at Triskelion (Utrecht, the Netherlands) by ultra-performance liquid chromatography (Ultimate 3000 UPLC; Thermo Scientific, Waltham, MA, USA). The UPLC unit was coupled to a high-resolution mass spectrometer (HR-MS; Q-Exactive mass spectrometer equipped with an electro-spray ionization probe; Thermo Scientific) and EDTA plasma samples (50 μl aliquots) were analyzed as described in detail previously [23]. The performance of the measurement unit was controlled throughout the analysis, i.e. by injecting 50 μl aliquots from a large (7 mL) reference EDTA plasma pool at regular intervals. Plasma biomarkers of inflammation and gut health were analyzed using ELISA following established protocols and optimized conditions [24,25]. More specifically, the following ELISAs purchased from R&D Systems (Abingdon, UK) were used: high-sensitive C-reactive protein (CRP; μg/ml) (D1707); serum amyloid A (SAA; μg/ml) (D3019), haptoglobin (μg/ml) (D8465-05) and lipopolysaccharide binding protein (LBP; μg/ml) (DY870-05).

### 2.5 DNA isolation

Feces samples were collected 4 weeks before surgery, and 6, and 24 months after surgery and were stored at −80 °C. For DNA isolation, fecal samples were thawed on ice and lysed by bead beating (mini-BeadBeater-24, Biospec Products Bartesville, USA) for 2 min at 2800 oscillations per minute in the presence of 800 μl of lysis buffer (Dneasy 96 Powersoil Pro QIAcube HT kit, Qiagen, Hilden, Germany), 500 μl zirconium beads (0.1 mm; Biospec products, Bartlesville, OK, USA). DNA was extracted using the Dneasy 96 Powersoil Pro QIAcube HT kit (Qiagen) in accordance with the manufactures recommendations. DNA quality was assessed by routine gel electrophoresis as well as by capillary electrophoresis on the Fragment Analyzer (Advanced Analytical, Heidelberg, Germany).

### 2.6 Amplicon sequencing

Changes in microbiota composition were analyzed using 16S rDNA amplicon sequencing. The V4 hypervariable region was targeted. 100 pg of DNA was amplified as described elsewhere [26] with the exception that 30 cycles were used instead of 35, applying F515/R806 primers [27]. Primers included Illumina adapters and a unique 8-nt sample index sequence key [26]. The amplicon libraries were pooled in equimolar amounts and purified using the QIAquick Gel Extraction Kit (QIAGEN). Amplicon quality and size were analyzed on a Fragment Analyzer (Advanced Analytical Technologies, Inc.). Paired-end sequencing of amplicons (approximately 400 base pairs) was conducted on the Illumina MiSeq platform (Illumina, Eindhoven, The Netherlands).

Sequence pre-processing, analysis and classification was performed using the DADA2 (version 1.14) software package in R [28]. Chimeric sequences were identified and removed using the ‘removeBimeraDenovo’ function from DADA2. The nonchimeric amplicon sequence variants (ASVs) were taxonomically classified using the assignTaxonomy function against the Silva nr 138 reference database. Taxonomic classification was performed up to the genus level.

### 2.7 IBS-SSS questionnaire

The IBS-SSS questionnaire is designed to monitor the disease progress and its treatment [29]. Here, it was used to assess the severity of abdominal pain after RYGB in the last ten days. The questionnaire consists of five questions concerning abdominal pain or discomfort, which each can be scored between 0 and 100 based on visual analogue scales. A total score below 75 indicates no discomfort, a score of 75–175 mild discomfort, a score of 175–300 moderate discomfort and a score over 300 severe discomfort. In this study we combined the moderate and severe categories, to make equally distributed groups.

### 2.8 Statistical analysis

Statistical analysis and visualization utilized R version 4.2.1. The ‘tidyverse’ package [30] facilitated data manipulation, while ‘ggplot2’ [31] was used to generate all plots. For the linear mixed-effects models, we applied the ‘lmerTes’ package to log-transformed chemistry data, considering timepoint as a fixed effect and subject as a random effect. The ‘emmeans’ package (v1.8.5; CRAN - Package emmeans (r-project.org)) enabled post-hoc pairwise comparisons. Spearman's rank correlation tests were performed using the ‘rstatix’ package.

Microbiome data processing utilized the ‘phyloseq’ package [32], filtering taxa based on prevalence and relative abundance using the method described elsewhere [33]. Differential expression analysis was conducted via ‘DESeq2’ [34], with results visualized in heatmaps. Before differential expression analysis, the continuous variables were first log-transformed, scaled and mean-centered.

Redundancy Analysis (RDA) was performed using the ‘vegan’ package (v2.6–4; CRAN - Package vegan (r-project.org)), after applying centered log-ratio transformation to the count data. Microbial diversity, assessed through the Shannon index using ‘vegan’, was further examined with linear mixed-effects models to explore timepoint-based changes. Microbial diversity was calculated on the raw count data so that no taxonomic filtered was applied.

Questionnaire data underwent polychoric correlation analysis using the ‘psych’ package (v2.3.6; CRAN - Package psych (r-project.org) to explore item relationships to obtain insights into response patterns.

## 3 Results

### 3.1 Descriptive statistics

In total 65 participants, of which 86.2 % female, were included in our analysis. Participant characteristics are listed in Table 1. Mean age of the participants was 46.2 ± 5.7 years with a mean BMI of 41.2 ± 3.6 kg/m^2^ and a mean WC of 123.9 ± 11.3 cm. Mean percentage of TWL was 25.7 ± 5.1 6 months and 33.7 ± 7.2 24 months after surgery. Finally, blood pressure, as well as medication use for hypertension significantly reduced after surgery.

**Table 1:** Characteristics of participants n = 65.

	Baseline	6 months after RYGB	24 months after RYGB	*p*-value
**Age, mean ± SD, years**	46.2 **±** 5.7			n/a
**Seks designated at birth, female, n (%)**	56 (86.2)			n/a
**Height, mean ± SD, m**	1.71 ± 0.07			n/a
**Weight, mean ± SD, kg**	121.1 ± 14.4	89.5 ± 14.4	80.1 ± 3.6	<0.001
**BMI, mean ± SD, kg/m**^**2**^	41.2 ± 3.6	30.4 ± 3.5	27.2 ± 3.4	<0.001
**WC**^a^**, mean ± SD, cm**	123.9 ± 11.3	100.9 ± 12.0	94.8 ± 10.8	<0.001
**TWL, mean ± SD, %**		25.7 ± 5.1	33.7 ± 7.2	<0.001
**Level of education**^b^**, n (%)**				
Low	4 (6.2)	n/a	n/a	n/a
Middle	38 (58.5)	n/a	n/a	n/a
High	23 (35.4)	n/a	n/a	n/a
**Medication use, n (%)**				
Oral antidiabetics	5 (7.7)	3 (4.6)	1 (1.6)	0.050
Insulin therapy	1 (1.5)	1 (1.5)	1 (1.5)	1.000
Blood pressure lowering agents	21 (32.2)	13 (20.0)	10 (15.4)	<0.001
Lipid lowering agents	11 (16.9)	7 (10.8)	8 (12.3)	0.074
**Current smoker, n (%)**	3 (4.6)	4 (6.2)	4 (6.2)	0.779
**Consuming alcohol**^c^**, n (%)**	25 (38.5)	15 (23.1)	27 (41.5)	0.003
Alcohol consumption, median (IQR), units per week	4.5 (5.3)	2.0 (3.0)	4.0 (3.8)	0.021
**Blood pressure**^d^**, mean ± SD, mm Hg**				
Systolic	138.7 ± 17.2	126.5 ± 16.9	129.2 ± 18.3	<0.001
Diastolic	85.3 ± 8.9	81.4 ± 10.3	79.7 ± 12.1	<0.000
**IBS-SSS, n (%)**				
None	n/a	n/a	18 (27.7)	n/a
Mild	n/a	n/a	26 (40.0)	n/a
Moderate to severe	n/a	n/a	21 (32.3)	n/a

Repeated measures analyses of variance, Cochran Test, or Friedman test were conducted to examine changes in characteristics over time. Significant changes over time are indicated by underscored p-values.

a Complete data on all timepoints is available for 48 participants.

b Verhage score ≤4 is defined a low level of education, a Verhage score of 5 as middle, a Verhage score of 6 or 7 as high [35].

c Complete data on all timepoints is available for 64 participants.

d Complete data on all timepoints is available for 49 participants Abbreviations: BMI = body mass index, WC = waist circumference, TWL = total weight loss, IBS-SSS = Irritable Bowel Syndrome Severity Scoring System, SD = standard deviation, IQR = inter quartile range.

### 3.2 Gastrointestinal complaints

The results of the IBS-SSS questionnaire are listed in Table 1. The IBS-SSS questionnaire revealed that 18 (27.7 %) participants had no gastrointestinal complaints, 26 (40.0 %) participants had mild complaints, and 21 (32.3 %) participants had moderate to severe complaints (of which 19 (29.2 %) had moderate, and 2 (3.1 %) had severe complaints). Differences in patient characteristics between the gastrointestinal complaint groups are listed in Table 2. The percentage of females was significantly different between groups, while body weight, BMI, WC, TWL, medication use, smoking, alcohol consumption and blood pressure did not differ between groups.

**Table 2:** Characteristics of participants for every gastrointestinal complaint group.

	Gastrointestinal complaints			
	None (n = 18)	Mild (n = 26)	Moderate – severe (n = 21)	p-value
**Age, mean ± SD, years**	47.6 ± 5.8	45.3 ± 5.6	46.0 ± 6.6	0.467
**Seks designated at birth, female, n (%)**	12 (66.7)	24 (92.3)	20 (95.2)	0.018
**Weight, mean ± SD, kg**	84.2 ± 13.4	77.1 ± 10.4	80.2 ± 11.0	0.143
**BMI, mean ± SD, kg/m**^**2**^	27.4 ± 3.4	26.4 ± 2.8	28.0 ± 4.0	0.267
**WC**^a^**, mean ± SD, cm**	98.2 ± 11.3	91.9 ± 10.4	95.3 ± 10.6	0.181
**TWL, mean ± SD, %**	32.9 ± 7.5	33.9 ± 7.1	34.1 ± 7.4	0.874
**Medication use, n (%)**				
Oral antidiabetics	0 (0)	1 (3.8)	0 (0)	0.467
Insulin therapy	0 (0)	1 (3.8)	0 (0)	0.467
Blood pressure lowering agents	4 (22.2)	5 (19.2)	1 (4.8)	0.251
Lipid lowering agents	3 (16.7)	2 (7.7)	3 (14.3)	0.636
**Current smoker, n (%)**	0 (0)	3 (11.5)	1 (4.8)	0.279
**Consuming alcohol**^b^**, n (%)**	8 (44.4)	13 (50.0)	6 (28.6)	0.319
Alcohol consumption, median (IQR), units per week	4.0 (4.0)	2.0 (2.5)	3.0 (2.5)	0.137
**Blood pressure, mean ± SD, mm Hg**				
Systolic	132.9 ± 16.2	122.2 ± 13.2	133.6 ± 22.6	0.076
Diastolic	81.4 ± 16.7	75.8 ± 9.8	82.3 ± 9.2	0.174
Occurrence of internal herniation^c^, n (%)	0 (0)	1 (3.8)	1 (4.8)	0.663

Chi-square, univariate analyses of variance and Kruskal-Wallis test were conducted to examine differences in characteristics between the gastrointestinal complaints groups.

a Data is available for 61 patients.

b Kruskal-Wallis test.

c The internal herniations were resolved more than 12 months before the time point analyzed in this study. Abbreviations: BMI = body mass index, WC = waist circumference, TWL = total weight loss, SD = standard deviation, IQR = inter quartile range.

### 3.3 Change in microbiota

**Fig. 2:**
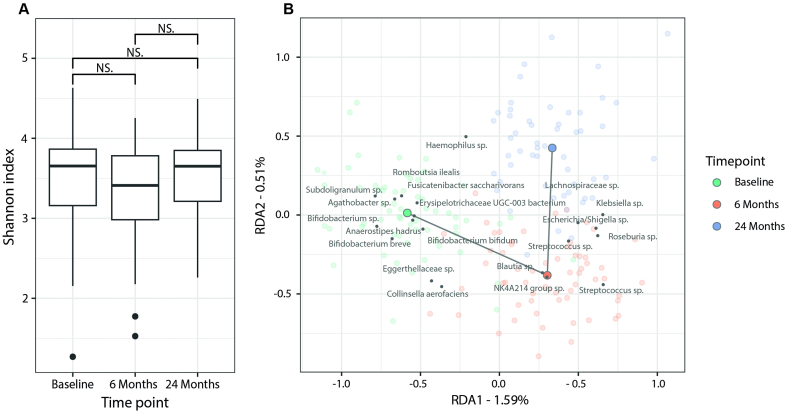
**Microbial diversity and composition**. A) No significant differences in microbiota alpha diversity (Shannon diversity) were found 6 and 24 months after Roux-en-Y gastric bypass surgery. B) Redundancy analysis (RDA) of microbiota beta diversity. At baseline a higher abundance of Bifidobacterium sp., and a lower abundance of Streptococcus sp. and Blautia were found compared to 6 months after surgery. 24 months after surgery Klebsiella sp. and Escherichia/Shigella sp. were more abundant compared to 6 months after surgery. Green dots represent the samples at baseline, orange dots those at 6 months after surgery and blue dots represent the samples that are present at 24 months after surgery.

Changes in gut microbiota after RYGB are presented in Fig. 2. No differences in alpha diversity were observed, while beta diversity significantly differed after surgery (Fig. 2, Table A1, see Supplementary file A). The shift in microbiota composition from baseline to 6 months after surgery was approximately 3 times more pronounced compared to the shift from 6 to 24 months after surgery (Fig. 2), indicating that the largest changes in composition occurred in the first 6 months after surgery. In particular, the contents of *Prevotella* sp., *Enterobacteriaceae* sp., *Fusobacterium*, *Akkermansia* sp., *Bacteroides* sp., *E. coli* and *Veillonella atypica* were higher, while *Faecalibacterium prausnitzii*, *Bifidobacterium* sp., *Lactobacillus*, *Citrobacter* sp., and *Coprococcus* sp. contents were lower 6 months after RYGB. The abundance of these species did not change from 6 to 24 months after surgery. In addition to these species, the abundance of *Negativibacillus* sp., *Alloprevotella* and *Peptoclostridium* sp. was significantly lower, whereas the abundance of *Catenibacterium*, *Gemella* sp. and *Blautia* sp. was significantly higher from 6 to 24 months after surgery. Moreover, the abundancy of aerobic bacteria was higher, while anaerobic bacteria showed lower counts after surgery. More specifically, anaerobic species such as *Bacteroides*, *Actinomyces graevenitzii* and *Bifidobacterium* were lower and aerobic species such as *E. coli* and *Klebsiella* were higher following RYGB.

### 3.4 Change in metabolites

**Fig. 3:**
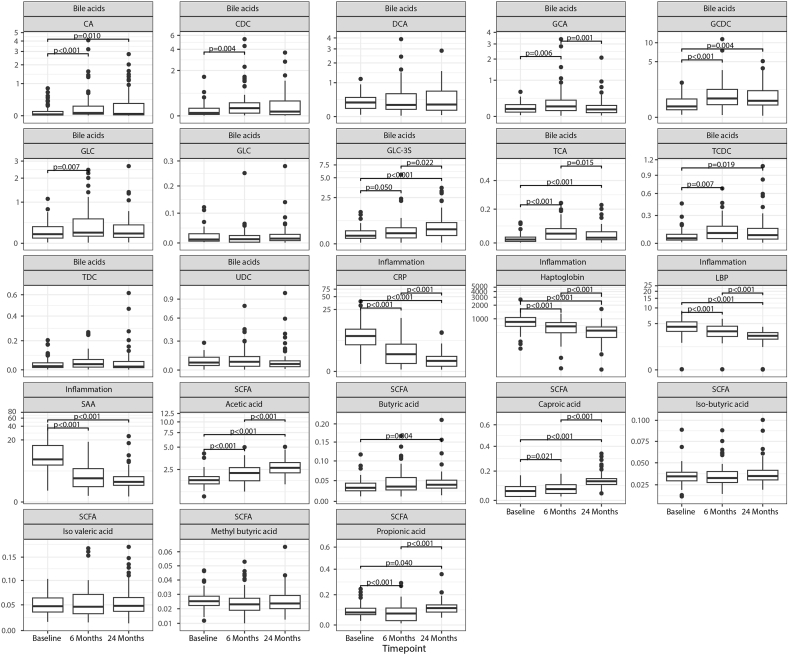
**Metabolite concentrations before and after Roux-en-Y gastric bypass surgery**. Differences in circulating bile acids, short-chain fatty acids and inflammatory markers before, and 6, and 24 months after surgery. Units of bile acids are: μmol/L, for SCFA: μg/ml and for inflammatory markers: μg/ml. Abbreviations: CA = cholic acid, CDC = chenodeoxycholic acid, DCA = deoxycholic acid, GCA = glycocholic acid, GCDC = glycochenodeoxycholic acid, GDC = glycodeoxycholic acid, GLC = glycolithocholic acid, GLC-3S = glycolithocholic acid-3-sulphate, TCA = taurocholic acid, TCDC = tauro-chenodeoxycholic acid, TDC = tauro-deoxycholic acid, UDC = ursodeoxycholic aci, CRP = C-reactive protein, LPB = lipopolysaccharide binding protein, SAA = serum amyloid A, SCFA = short-chain fatty acid.

Bile acids, SCFA and inflammatory markers were analyzed before and after surgery (Fig. 3, Table A2, Table A3, see Supplementary file A). Several bile acid concentrations changed after RYGB. Compared to baseline, CDC and GDC levels were significantly higher 6 months after surgery (p = 0.004, p = 0.007), albeit no changes were found 24 months after surgery or between 6 months and 24 months after surgery. CA, GCDC, and TCDC levels were significantly higher 6 months after surgery (p < 0.001, p < 0.001, p = 0.007), and slightly, but not significantly lower, 24 months after surgery, remaining significantly higher compared to baseline (p = 0.010, p = 0.004, p = 0.019). TCA levels were significantly higher 6 months after surgery (p < 0.001), and its levels significantly dropped 24 months after surgery (p = 0.015), albeit remaining significantly higher compared to baseline (p < 0.001). GCA levels were significantly higher 6 months after surgery (p = 0.006) but returned to baseline level 24 months after surgery (p = 0.001). GLC-3S was higher 6 months (p = 0.050) and 24 months after surgery compared to baseline (p < 0.001). No significant differences were found for DCA, GLC, TDC, UDC after RYGB. SCFA levels also changed after surgery. Compared to baseline, acetic acid and caproic acid were significantly higher 6 (p < 0.001, p = 0.021) and 24 months after surgery (p < 0.001, p < 0.001), while butyric acid was only significantly higher 24 months after surgery (p = 0.004). Compared to baseline, propionic acid was significantly lower 6 months (p < 0.001), but significantly higher 24 months after surgery (p = 0.040). No significant differences were observed for iso-butyric acid, methyl butyric acid and iso valeric acid. The inflammatory markers CRP, haptoglobin and LBP were significantly lower 6 months after surgery (p < 0.001, p < 0.001, p < 0.001) and continued to decrease 24 months after RYGB (p < 0.001, p < 0.001, p < 0.001). SAA was significantly lower 6 months after surgery (p < 0.001), and remained stable 24 months after surgery.

### 3.5 Link between microbiota and gastrointestinal complaints

**Fig. 4:**
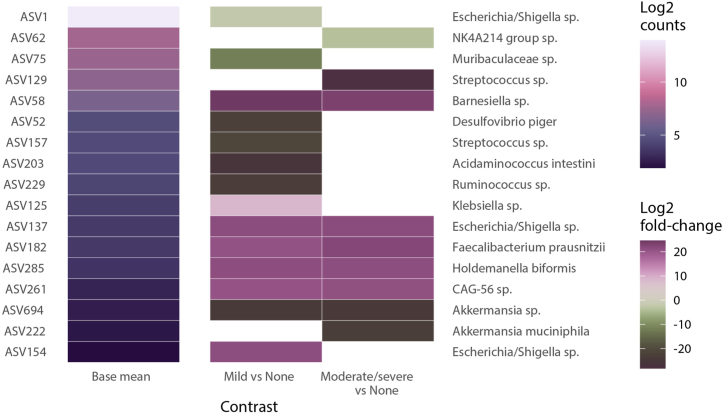
**Heatmap of differential abundance of the association between bacterial taxa and gastrointestinal complaints**. The ‘base mean’ column displays the average abundance of bacterial taxa in the cohort, with colors corresponding to the ‘Log2 counts' legend. The subsequent columns compare bacterial abundance in individuals with mild or moderate to severe complaints to those with none (no complaints), indicated by the ‘Log2 fold-change’ legend. A positive Log2 fold-change signifies increased abundance associated with complaints, while a negative value indicates decreased abundance. Abbreviations: ASV = Amplicon Sequence Variant.

The relationship between microbiota and severity of gastrointestinal complaints 24 months after surgery is depicted in Fig. 4. No significant correlations were found between the microbiota alpha diversity and severity of gastrointestinal complaints 24 months after RYGB (Table A4, see Supplementary file A). However, several bacterial species significantly correlated with the severity of gastrointestinal complaints (Fig. 4). Amongst others, *Barnesiella* sp., *Escherichia*/*Shigella* sp., *Faecalibacterium prausnitzii* and *Holdemanella biformis* showed a positive correlation with mild and moderate to severe gastrointestinal complaints. In contrast, *Muribaculaceae* sp., *Desulfovibrio piger*, *Streptococcus* sp., *Acidaminococcus intestini*, *Ruminococcus* sp. showed an inverse correlation with mild gastrointestinal complaints. In addition, *Akkermansia* sp. correlated inversely with mild and moderate to severe gastrointestinal complaints while *Akkermansia muciniphila* and *NK4A214 group sp*. only correlated inversely with moderate to severe gastrointestinal complaints.

### 3.6 Link between metabolites and gastrointestinal complaints

**Fig. 5:**
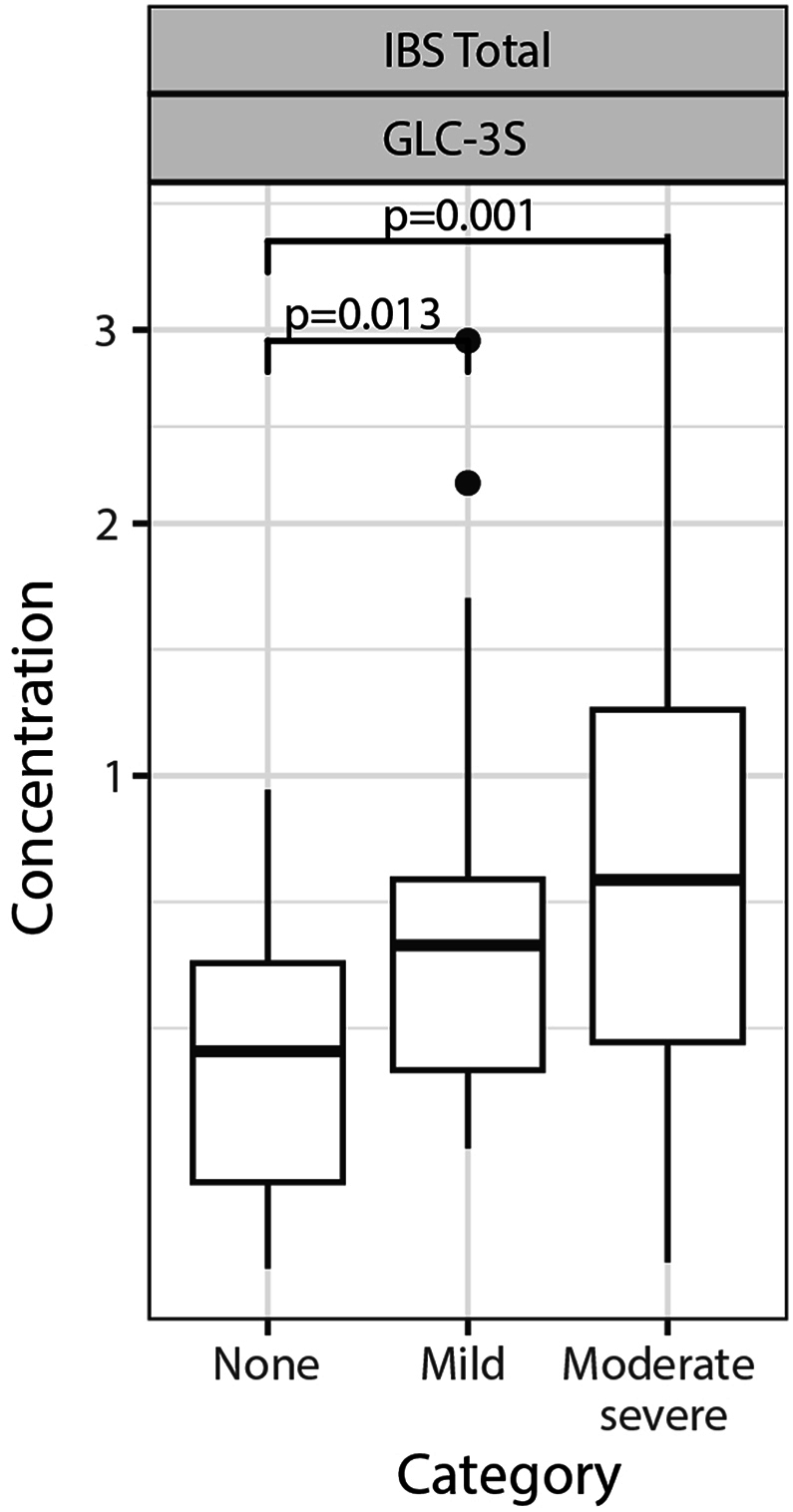
**Correlation between GLC-3S and severity of gastrointestinal complaints.** GLC-3S is significantly higher in participants with mild and moderate to severe gastrointestinal complaints. Abbreviations: GLC-3S = glycolithocholic acid-3-sulphate, IBS = Irritable Bowel Syndrome.

The difference in metabolite concentration between the participants with and without gastrointestinal complaints is depicted in Fig. 5. We found that the concentration of GLC-3S was significantly higher in subjects with mild (p = 0.013) and moderate to severe gastrointestinal complaints (p = 0.001). SCFA and inflammatory markers did not show significant differences between groups (Table A5, see Supplementary file A).

### 3.7 Link between metabolites and microbiota 24 months after surgery

**Fig. 6:**
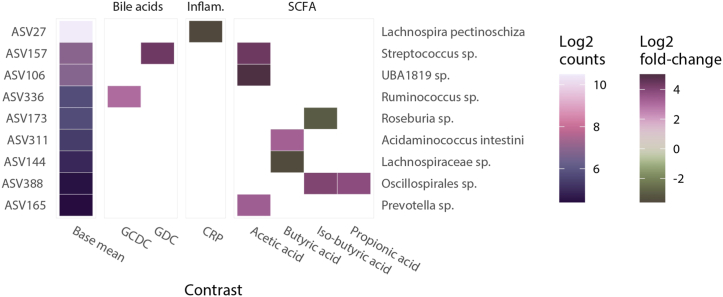
**Heatmap of differential abundance of the association between metabolites and microbial taxa.** GDC, GCDC, acetic acid and propionic acid showed positive associations, while CRP showed a negative association with the identified taxa. Butyric acid and iso-butyric acid showed both positive and negative associations. The ‘base mean’ column shows the average abundance of the given bacterial taxon in the whole cohort. The ‘base mean’ colors correspond to the ‘Log2 counts’ legend. The other columns show the associations between metabolites and bacterial taxa. The colors for the differential abundances correspond to the Log2 fold-change legend. A positive Log2 fold-change indicates a positive association and a negative log2fold-change indicates a negative association. Abbreviations: GCDC = glycochenodeoxycholic acid, GDC = glycodeoxycholic acid, CRP = C-reactive protein, ASV = Amplicon Sequence Variant.

The relationship between metabolites and microbiota alpha and beta diversity is visible in Fig. 6. No significant (p < 0.01) correlations were found between microbiota alpha diversity and metabolites (Table A6, see Supplementary file A). However, significant correlations between metabolites and microbiota beta diversity were found (Fig. 6, Table A7, see Supplementary file A). GDC (p = 0.018), GCDC (p = 0.042), acetic acid (p = 0.029, p = 0.006, p = 0.029) and propionic acid (p = 0.008) showed positive correlations, while CRP showed a negative correlation with beta diversity (p = 0.004). Butyric acid correlated positively with *Acidaminococcus intestine* (p = 0.027) and inversely with *Lachnospiraceae* sp. (p = 0.043). Iso-butyric acid correlated positively with *Oscillospirales* sp. (p = 0.008) and inversely with *Roseburia* sp. (p = 0.034). GLS-3S, the metabolite increased in subjects with gastrointestinal complaints, did not show a significant relationship with microbiota beta diversity.

## 4 Discussion

In this study the relation between gastrointestinal complaints, microbiota diversity and its metabolites was assessed after RYGB. According to the IBS-SSS questionnaire, 40.0 % of the participants had mild complaints and 32.3 % of the participants had moderate to severe gastrointestinal complaints 24 months after surgery. Microbiota beta diversity significantly changed at all timepoints, with the highest change from baseline to 6 months after surgery, although the alpha diversity was comparable over time. Bile acids and SCFA plasma concentrations were significantly higher, and inflammatory markers were significantly lower after surgery. Microbiota beta diversity significantly correlated with mild and moderate to severe gastrointestinal complaints, while no correlation between alpha diversity and gastrointestinal complaints was found 24 months after surgery. Moreover, patients with gastrointestinal complaints showed higher levels of GLC-3S. Finally, several correlations were found between genera of the gut microbiota and plasma metabolites. Although, GLS-3S did not show a significant relationship with the specific microbiota genera.

Our results show that 32.3 % of the patients suffered from moderate to severe gastrointestinal complaints after RYGB. This result is in line with previous literature demonstrating the presence of abdominal pain after MBS in 30 % of the patients [3], indicating that our study population is a representative MBS patient population with gastrointestinal complaints.

In agreement with previous studies we could not detect differences in microbial alpha diversity [36,37], but microbiota beta diversity significantly differed after RYGB [9,13,14]. This change in microbiota beta diversity was most pronounced from baseline to 6 months, which could be an effect of the dietary changes early after surgery, as dietary intake is highly associated with microbiota composition [38]. Previously it has been demonstrated that the daily energy intake (kcal) and total fat intake showed a negative correlation with the percentage excess weight loss at 12 months after surgery. However, after 24 months this correlation was no longer significant [39]. These findings indicate that dietary intake changed from 12 to 24 months after surgery, which may also be the case in our cohort, potentially explaining the greatest change in microbiota composition 6 months after surgery. Specially, we found higher counts of *Prevotella*, *Bacteroides Enterobacteriaceae* sp., and *Fusobacterium*, as well as a lower counts of *Bifidobacterium* and *Faecalibacterium prausnitzii* after MBS. In contrast with literature, we found a higher abundance of *Blautia* 24 months after surgery [14]. Furthermore, we observed a change from anaerobic microbiota towards an aerobic microbiota after RYGB. This might be explained by anatomical changes of the gastrointestinal tract after surgery. The passage of nutrients changes and the pH of the digestive tract increases after RYGB, potentially creating a more favorable environment for aerobic bacteria [40]. These results indicate that RYGB indeed changes the gut microbiota composition, resulting in changes of the abundance of specific microorganisms. However, it is still unclear how these gut bacteria influence gastrointestinal health and whether direct or indirect mechanisms are involved. It can be speculated that direct mechanisms, via gut bacteria or bacterial fragments, activate nociceptors. Another possibility may be through indirect mechanisms, where gut bacteria alter the milieu of produced metabolites, which may affect metabolic inflammatory homeostasis (e.g. intestinal inflammation) and thereby affect pain sensation.

As MBS alters gut microbiota composition, a change in metabolite composition was also expected. In agreement with previous human studies [41], we found higher fasting plasma bile acid concentrations after RYGB. In addition, a cross-sectional study revealed that individuals who underwent RYGB, had higher plasma bile acid concentrations compared to weight-matched individuals [42]. It is suggested that this increase in bile acids after surgery might be involved in surgery related metabolic improvements (e.g. improved glucose and lipid metabolism), as bile acids can exert endocrine functions that regulate metabolism [41]. However, in our study some bile acids (GCA, TCA) showed fluctuations in concentration before, and 6, and 24 months after surgery. Previous longitudinal studies also demonstrated considerable fluctuations in plasma bile acids over time [41]. These changes in bile acid concentrations might have biological effects on lipid and glucose homeostasis and energy expenditure – in a more indirect way – and may be involved in the development of gastrointestinal complaints. However, underlying mechanisms remain unknown and regardless of what has been demonstrated clinically, current evidence is insufficient to make the case that bile acids themselves are causal to gastrointestinal complaints.

We found higher levels of acetic acid, propionic acid, caproic acid and butyric acids 6 and 24 months after surgery. Iso-butyric acid, methyl butyric acid and iso valeric acid did not change over time, suggesting an increased microbial saccharolytic fermentation specifically in our cohort. In contrast, other studies revealed that total SCFA levels remained stable or decreased after MBS [37,43,44]. These different results might be explained by the fact that we assessed SCFA in plasma samples instead of faecal samples. However, one previous study, who also investigated the effect of RYGB on plasma SCFA levels, demonstrated reduced plasma acetate, propionate, and butyrate levels and increased plasma iso-butyric acid and iso valeric acid levels 12 months after RYGB [45]. Thus, results on plasma SCFA levels after RYGB are conflicting, which might be an effect of preoperative support methods (e.g. diets). Nonetheless, more research is necessary to elucidate the effect of RYGB on plasma SCFA levels. Regarding plasma inflammatory markers, our results are in line with previous research showing that circulating inflammatory makers are lower after surgery [46,47]. This suggests that weight loss may have beneficial effects on inflammation.

We found significant positive correlations between microbial genera and the severity of gastrointestinal complaints. In our cohort, fecal levels of *Barnesiella* sp., *Holdemannella biformis*, and *Faecalibacterium prausnitzii* increased in subjects with mild and moderate to severe gastrointestinal complaints. Previously it has been shown that *Barnesiella intestinihominis* is positively associated with abdominal discomfort and pain [48], suggesting that higher levels of *Barnesiella* could induce gastrointestinal complaints. Evidence shows that *Holdemannella biformis* which has anti-inflammatory effects on colitis, is inversely correlated with IBS [49] and contributes to a healthy microbiome [50]. However, our cohort revealed that participants with gastrointestinal complaints had higher counts of *Holdemanella biformis*. Thus results are conflicting, suggesting that this bacterial species has different effects on gastrointestinal complaints after RYGB. *Faecalibacterium prausnitzii*, a bacteria involved in the fermentation of dietary fibers, is able to reduce visceral hypersensitivity by improving the intestinal epithelial barrier [51]. We showed that *F. prausnitzii* was positively correlated with gastrointestinal complaints. Nonetheless, it is important to keep in mind that *F. prausnitzii* cannot optimally digest fibers on its own, albeit in the presence of *Ruminococcus callidus* its functionality increases [4]. We also showed that participants with gastrointestinal complaints had lower counts of *Ruminococcus* and the abundance of *Ruminoccus* decreased after surgery, possibly explaining the negative effects of *F. prausnitzii* on gastrointestinal symptoms in our cohort.

Significant inverse correlations have also been found between microbial genera and the severity of gastrointestinal complaints. Lower faecal levels of *Ruminococcus* sp., *Akkermansia* sp., *Muribaculaceae* sp., *Desulfovibrio piger* and *NK4A214 group sp*. were observed in participants with gastrointestinal complaints. Similar to observations in patients with inflammatory bowel disease (IBD) [52], we found lower levels of *Ruminococcus* sp. in patients with mild gastrointestinal complaints, suggesting that *Ruminococcus* sp. has beneficial effects on gastrointestinal health. Administration of pasteurized *Akkermansia* downregulates inflammatory cytokine levels and thereby improves colon injury and diarrheal symptoms in antibiotic induced diarrhea in mice [53]. In our study, lower levels of *Akkermansia* have been found in participants with gastrointestinal complaints, indicating that lower levels of *Akkermansia* may induce intestinal inflammation and thereby cause gastrointestinal complaints after RYGB. There are conflicting results regarding the health benefits of *Muribaculaceae* sp., a SCFA producing bacteria. Increased levels of *Muribaculaceae* can suppress inflammation and ameliorate antibiotic induced intestinal damage in mice [54]. However, *Muribaculaceae* sp. was also associated with reduced mucin expression and increased intestinal permeability [55]. We found lower levels of *Muribaculaceae* in subjects with mild gastrointestinal complaints, suggesting that lower levels of *Muribaculaceae* after RYGB may increase inflammation and intestinal damage, leading to gastrointestinal complaints. The abundancy of *Desulfovibrio piger* is significantly higher in patients with IBD compared to healthy subjects [56]. Additionally, obese mice administered with *Desulfovibiro piger* showed decreased villus length and crypt depth in the ileum [57], which could cause stomach complaints. However, *Desulfovibrio* was also positively associated with beneficial genera and negatively associated with harmful genera [58]. Although previous results are conflicting, our study supports the beneficial effects of *Desulfovibrio piger*, as lower levels were found in subjects with mild gastrointestinal complaints. Finally, the *NK4A214 group sp*., which is closely related to *Oscillospiraceae* [59] was inversely correlated with moderate to severe gastrointestinal complaints*.* As *Oscillospiraceae* is a butyrate producing bacteria, and thus important for gut barrier function [60], we suggest that reduced levels of the *NK4A214 group sp*. may be associated with gastrointestinal complaints as a consequence of increased intestinal permeability. In summary, this study suggests that a change in microbial composition after RYGB may be involved in the development of gastrointestinal complaints.

Many metabolites serve as signaling molecules that affect biological functions. Altered metabolite production in the gut, either from the microbiota or host or their interaction, may induce gastrointestinal alterations, leading to gastrointestinal complaints [61]. Moreover, the composition of serum bile acids changes following MBS [62]. We indeed detected many changes in plasma metabolites, including increased bile acid concentrations after surgery. However, only one plasma metabolite was significantly higher in subjects with gastrointestinal complaints, namely the bile acid GLC-3S. Therefore, it is likely that GLC-3S is a reflection of a metabolic-inflammatory distortion that predisposes to the development of gastrointestinal pain. Moreover, GLC-3S is a secondary bile acid, suggesting that the modification from primary to secondary bile acids by the microbiota might be involved in the development of gastrointestinal complaints. Nonetheless, future studies are needed to assess whether GLC-3S coincides or precedes with gastrointestinal complaints.

Several correlations were found between the gut microbiota and its metabolites. Although, GLS-3S did not show a significant correlations with the specific microbiota genera associated with gastrointestinal complaints. This indicates that changes in GLS-3S are rather a reflection of activity than absolute number (16S counts) of microorganisms. It is noteworthy to mention that metabolites produced by the identified microorganisms may exert effects within the gut tissue that are causal to gastrointestinal complaints, even though they are not (or are below the detection limit) released in plasma. Amongst the significant correlations, acetic acid was positively associated with *Streptococcus* sp. In our cohort, patients with mild gastrointestinal complaints showed a lower abundance of *Streptococcus* sp. Previous studies show that SCFA, including acetic acid have beneficial effects on host physiology and could relieve constipation [63,64]. This suggests that subjects with lower *Streptococcus* sp. counts, might also have lower acetic acid levels and therefore may experience gastrointestinal symptoms. Nonetheless, it should be noted that metabolites are measured in plasma whereas the fecal gut microbiota is a reflection of the gut lumen.

### 4.1 Limitations

Our study has some limitations. First, we could only assess the severity of gastrointestinal complaints and not the type of complaints (e.g. obstipation-associated, diarrhea-associated). However, this study is still one of the first to investigate the relation between the gut microbiota, its metabolites and gastrointestinal complaints after RYGB using advanced quantitative metabolomics platforms to accurately measure bile acids, SCFA and inflammatory markers. Second, in this study the gastrointestinal complaints before surgery were not taken into account. This may be important as it has been shown that preoperative symptoms can be related to the severity of postoperative symptoms [6]. Third, we asked participants to fill out the IBS-SSS questionnaire at one time point, which may have induced recall-bias. However, the IBS-SSS questionnaire captures gastrointestinal complaints close to the time of experience, thereby reducing distortion from forgetting and recall bias, and obtaining reasonably accurate data. Fourth, our cohort did not have an equal sex distribution and the sex distribution between gastrointestinal complaint groups significantly differed. It is important to consider this unequal distribution as the development of gastrointestinal disorders is higher in women compared to men [65], indicating that our results could be biased by an unequal sex distribution. Fifth, the severity of gastrointestinal complaints can be multifactorial and the exact contribution of the microbiome and its metabolites to the IBS-SSS score cannot fully be assessed in observational studies and will always depend on a multiplicity of underlying factors including medication, diet, and lifestyle. Since all of these factors can also influence the microbiota, it may be possible in future studies to alter specific species of the microbiota and test strategies of postoperative microbiota manipulation to prevent or improve gastrointestinal complaints after RYGB.

### 4.2 Conclusion

In conclusion, our results show that approximately 30 % of the participant suffers from moderate to severe gastrointestinal complaints after RYGB. Moreover, we observed a significant difference in beta diversity after surgery, shifting from an anaerobic to a more aerobic environment two years after surgery. Using correlation analyses, we identified specific microbial genera, including *Muribaculaceae*, *Barnesiella*, *Desulfovibrio piger* and *Faecalibacterium prausnitzii* which were related to the severity of gastrointestinal complaints. Plasma metabolites also showed major changes after surgery: most plasma bile acids concentrations were higher, and plasma SCFA were higher, or did not change, while inflammatory markers were lower two years after surgery. A specific plasma metabolite (GLC-3S) was associated with gastrointestinal complaints, pointing to a potential use as a objective (early) biomarker and possible evaluation of causality. Altogether, these results indicate that the human intestinal microbiome, with respect to certain genera and associated metabolites might be involved in the development of gastrointestinal complaints after RYGB. Future studies should concentrate on establishing causality and mechanistic rationales how these gut bacteria alter the gut environment and whether microorganisms directly or indirectly (via their metabolites) can elicit gastrointestinal complaints. Such studies can contribute to the development of strategies to reduce the risk of gastrointestinal symptoms after MBS.

## CRediT authorship contribution statement

**Emma Custers:** Writing – original draft, Project administration, Methodology, Investigation, Conceptualization. **Yonta G.R. van der Burgh:** Writing – original draft, Methodology. **Debby Vreeken:** Writing – review & editing, Data curation. **Frank Schuren:** Writing – review & editing, Investigation. **Tim J. van den Broek:** Writing – review & editing, Formal analysis. **Lars Verschuren:** Writing – review & editing. **Ivo de Blaauw:** Methodology. **Mark Bouwens:** Writing – review & editing. **Robert Kleemann:** Writing – review & editing, Methodology, Funding acquisition. **Amanda J. Kiliaan:** Writing – review & editing, Supervision, Methodology, Funding acquisition, Conceptualization. **Eric J. Hazebroek:** Writing – review & editing, Supervision, Methodology.

## Availability of data and materials

All data generated or analyzed during this study are included in this published article (see Supplementary file B).

## Funding

This work was supported by a Rijnstate-10.13039/501100006209Radboudumc Promotion Fund grant. Plasma and fecal biomarker analyses were conducted with funds of 10.13039/501100019926TNO research programs ‘ERP-Body-Brain Interactions’,‘PMC-Functional Biomarkers’ and ‘GLoBAL-1 consortium (10.13039/501100019926Netherlands Organisation for Applied Scientific Research 10.13039/501100019926TNO, 10.13039/501100006209Radboud University Medical Center, Rijnstate Hospital, and Nordic Bioscience)’.

## Declaration of competing interest

The authors declare that they have no known competing financial interests or personal relationships that could have appeared to influence the work reported in this paper.
