# Supplementary material for: Gastrointestinal complaints after Roux-en-Y gastric bypass surgery. Impact of microbiota and its metabolites
Source: Heliyon. 2024 Oct 31;10(21):e39899. doi: 10.1016/j.heliyon.2024.e39899 (PMC11570293; doi:10.1016/j.heliyon.2024.e39899)
Supplement: Multimedia component 1 [file mmc1.docx]

**IBS-SSS vragenlijst (for english see below)**

**Instructie:** Deze vragen gaan over de ernst van uw klachten. Beantwoord de volgende vragen zoals u de afgelopen 10 dagen klachten heeft ervaren. Voor elke vraag kunt u een score van 0 tot en met 10 geven, het cijfer mag u in het vierkant opschrijven.

1. **Hoeveel last had u de afgelopen 10 dagen van buikpijn?**Pijnscore: Op een schaal van 0 tot en met 10.
   0 = geen pijn 10 = ondragelijke pijn.
2. **Hoeveel dagen (in een periode van 10 dagen) had u buikpijn?**

Aantal dagen, tussen 0 en 10 dagen

1. **Hoe veel last had u de afgelopen 10 dagen van een opgeblazen gevoel?**

Pijnscore: Op een schaal van 0 tot en met 10.
0 = geen pijn 10 = ondragelijke pijn.

1. **Hoe tevreden was u de afgelopen 10 dagen met uw stoelgang?**

Score voor tevredenheid: Op een schaal van 0 tot en met 10.
0 = tevreden 10 = zeer ontevreden

1. **Hoeveel hinder had u de afgelopen 10 dagen van uw buikklachten op het dagelijks leven?**

0= geen hinder, 10 = ondragelijk

**IBS-SSS questionnaire in English**

**Instruction:** These questions concern the severity of your symptoms. Please answer the following questions as you have experienced symptoms in the past 10 days. For each question you can give a score from 0 to 10, you may write the number in the box.

1. **How much did you experience abdominal pain in the past 10 days?**Pain score: On a scale from 0 to 10.
   0 = no pain 10 = unbearable pain.
2. **How many days (in a 10-day period) did you have abdominal pain?**

Number of days, between 0 and 10 days.

1. **In the past 10 days, how much did you suffer from bloating?**

Pain score: On a scale from 0 to 10.
0 = no pain 10 = unbearable pain.

1. **In the past 10 days, how satisfied were you with your bowel movements?**

Satisfaction score: On a scale from 0 to 10.
0 = satisfied 10 = very dissatisfied

1. **In the past 10 days, how much did your abdominal discomfort interfere with your daily life?**

0= no hindrance, 10 = unbearable
