# Supplementary material for: Gastrointestinal complaints after Roux-en-Y gastric bypass surgery. Impact of microbiota and its metabolites
Source: Heliyon. 2024 Oct 31;10(21):e39899. doi: 10.1016/j.heliyon.2024.e39899 (PMC11570293; doi:10.1016/j.heliyon.2024.e39899)
Supplement: Multimedia component 2 [file mmc2.docx]

Supplementary results

**Table A1.** Change in microbiota beta diversity 6 and 24 months post-surgery

| Bacteria | RDA1 | RDA2 |
| --- | --- | --- |
| Subdoligranulum sp. | -0.402 |  |
| Bifidobacterium sp. | -0.396 |  |
| Bifidobacterium breve | -0.35 |  |
| Agathobacter sp. | -0.344 |  |
| Klebsiella sp. | 0.336 |  |
| Streptococcus sp. | 0.334 | -0.164 |
| Roseburia sp. | 0.324 |  |
| Romboutsia ilealis | -0.314 |  |
| Escherichia/Shigella sp. | 0.31 |  |
| Erysipelotrichaceae UCG-003 bacterium | -0.286 |  |
| Anaerostipes hadrus | -0.285 |  |
| Fusicatenibacter saccharivorans | -0.268 |  |
| Bifidobacterium bifidum | -0.254 |  |
| Lachnospiraceae sp. | 0.252 |  |
| Eggerthellaceae sp. | -0.233 | -0.147 |
| Lachnospiraceae sp. | -0.231 |  |
| Streptococcus sp. | 0.228 |  |
| Ruminococcus sp. | -0.227 |  |
| Clostridium sensu stricto 1 perfringens | 0.227 |  |
| Faecalibacterium prausnitzii | -0.225 |  |
| Lachnospiraceae sp. | 0.215 |  |
| Enterobacteriaceae sp. | 0.211 |  |
| Bacteroides massiliensis | 0.208 |  |
| Bifidobacterium sp. | -0.201 |  |
| Ruminococcus bromii | -0.192 |  |
| Bacteroides vulgatus | 0.187 |  |
| Blautia massiliensis | -0.186 |  |
| Faecalibacterium prausnitzii | -0.184 | 0.0802 |
| Collinsella aerofaciens | -0.181 | -0.176 |
| Romboutsia sedimentorum | -0.179 |  |
| Lachnospiraceae sp. | 0.178 |  |
| Bacteroides sp. | 0.178 |  |
| Dialister invisus | -0.176 |  |
| Holdemanella sp. | -0.174 |  |
| Christensenellaceae R-7 group sp. | -0.173 |  |
| Alistipes indistinctus | -0.165 |  |
| Intestinimonas sp. | 0.164 |  |
| Sutterella massiliensis | -0.163 |  |
| Blautia sp. | 0.163 |  |
| NK4A214 group sp. | 0.163 |  |
| Agathobacter sp. | -0.162 |  |
| Intestinibacter bartlettii | -0.162 |  |
| Bifidobacterium dentium | -0.161 |  |
| Alistipes shahii | 0.159 |  |
| Incertae Sedis sp. | 0.159 |  |
| Ruminococcus sp. | -0.157 |  |
| NK4A214 group sp. | 0.154 | -0.151 |
| Bacteroides uniformis | -0.154 |  |
| Agathobacter sp. | -0.153 | 0.0973 |
| Clostridium sensu stricto 1 sp. | -0.151 |  |
| Coprococcus sp. | -0.148 |  |
| Streptococcus sp. | 0.144 |  |
| Bacteroides coprocola | -0.143 |  |
| Veillonella atypica | 0.143 |  |
| Lachnospiraceae sp. | 0.14 |  |
| Faecalibacterium cf. | -0.139 |  |
| Blautia sp. | 0.138 | -0.144 |
| Lachnospiraceae sp. | -0.134 | -0.1 |
| Akkermansia muciniphila | 0.134 |  |
| Blautia faecis | 0.133 |  |
| Lactobacillus sp. | -0.133 |  |
| UCG-002 sp. | 0.133 |  |
| Akkermansia sp. | 0.132 |  |
| Alistipes finegoldii | 0.129 |  |
| Lachnoclostridium sp. | 0.128 |  |
| Bacteroides caccae | 0.128 |  |
| Escherichia/Shigella sp. | 0.127 |  |
| Citrobacter sp. | 0.127 | -0.108 |
| Solobacterium sp. | -0.126 |  |
| Actinomyces graevenitzii | -0.126 |  |
| Akkermansia muciniphila | 0.126 | -0.093 |
| Marvinbryantia sp. | 0.125 |  |
| Mogibacterium sp. | 0.125 | 0.116 |
| Paraprevotella clara | -0.125 | 0.0863 |
| Desulfovibrio piger | 0.122 |  |
| Streptococcus sp. | -0.122 |  |
| Coprococcus comes | -0.121 |  |
| Subdoligranulum sp. | -0.12 |  |
| Alistipes sp. | 0.12 |  |
| Prevotella copri | 0.119 |  |
| Faecalibacterium sp. | -0.118 |  |
| Dialister sp. | -0.117 |  |
| Peptococcus sp. | -0.117 |  |
| Howardella ureilytica | -0.113 |  |
| Turicibacter sanguinis | 0.109 |  |
| Roseburia sp. | 0.108 |  |
| Streptococcus sp. | 0.106 |  |
| Lachnospira pectinoschiza | 0.103 |  |
| Streptococcus sp. | 0.103 |  |
| Muribaculaceae sp. | 0.102 |  |
| Haemophilus sp. | -0.1 | 0.185 |
| Anaerostipes sp. | 0.1 |  |
| Prevotella sp. | 0.0987 |  |
| Klebsiella sp. | 0.0986 |  |
| NK4A214 group sp. | 0.0962 | 0.109 |
| Prevotella sp. | 0.096 |  |
| Morganella morganii | -0.095 |  |
| UCG-005 sp. | 0.0938 |  |
| Enterobacteriaceae sp. | 0.092 |  |
| Akkermansia sp. | 0.086 |  |
| Pluralibacter sp. | 0.0858 |  |
| Prevotella sp. | 0.0848 |  |
| Escherichia/Shigella coli | 0.0837 |  |
| Fusobacterium sp. | 0.0834 |  |
| Catenisphaera sp. | -0.0808 |  |
| Acidaminococcus intestini | 0.0788 |  |
| Akkermansia sp. | 0.0787 | 0.112 |
| Subdoligranulum sp. |  | 0.127 |
| Oscillospirales sp. |  | 0.11 |
| Lachnospiraceae sp. |  | 0.122 |
| Bacteroides intestinalis |  | -0.146 |
| Blautia sp. |  | 0.0844 |
| Peptoclostridium sp. |  | -0.118 |
| Prevotella sp. |  | -0.0514 |
| Negativibacillus sp. |  | -0.112 |
| Parabacteroides johnsonii |  | -0.143 |
| Gemella sp. |  | 0.13 |
| Monoglobus pectinilyticus |  | 0.106 |
| Alloprevotella sp. |  | -0.141 |
| Clostridia UCG-014 sp. |  | -0.107 |
| Lachnospiraceae sp. |  | 0.0998 |
| Catenibacterium sp. |  | 0.158 |
| Prevotella sp. |  | 0.126 |
| Marvinbryantia sp. |  | -0.146 |
| Colidextribacter massiliensis |  | -0.137 |
| Lachnospiraceae ND3007 group sp. |  | 0.117 |
| Bilophila wadsworthia |  | -0.156 |
| Lachnospiraceae sp. |  | 0.0722 |
| Streptococcus sp. |  | 0.106 |
| Bacteroides dorei |  | -0.144 |

*RDA1 indicates the change from baseline to 6 months, were a negative RDA indicates that the abundance is lower at 6 months vs baseline. RDA 2 indicates the change from 6 months to 24 months, were a negative RDA indicates a lower abundance at 24 months vs 6 months.*

**Table A2.** Main effect of change in metabolites before and after bariatric surgery

| Type | Variable | Statistic | p-value |
| --- | --- | --- | --- |
| Inflammation | CRP | 207 | <0.001 |
| Inflammation | SAA | 220 | <0.001 |
| Inflammation | Haptoglobin | 79.5 | <0.001 |
| Inflammation | LPS | 192 | <0.001 |
| Bile acids | CA | 12.7 | 0.002 |
| Bile acids | CDC | 8.69 | 0.013 |
| Bile acids | DCA | 1.24 | 0.539 |
| Bile acids | GCA | 12.5 | 0.002 |
| Bile acids | GCDC | 19.9 | <0.001 |
| Bile acids | GDC | 7.44 | 0.024 |
| Bile acids | GLC | 1.68 | 0.431 |
| Bile acids | GLC-3S | 18.8 | <0.001 |
| Bile acids | TCA | 45.4 | <0.001 |
| Bile acids | TCDC | 8.93 | 0.012 |
| Bile acids | TDC | 2.12 | 0.347 |
| Bile acids | UDC | 2.97 | 0.227 |
| SCFA | Acetic acid | 74.8 | <0.001 |
| SCFA | Propionic acid | 32.7 | <0.001 |
| SCFA | Butyric acid | 8.67 | 0.013 |
| SCFA | Iso-butyric acid | 3.27 | 0.195 |
| SCFA | Methyl butyric acid | 1.1 | 0.577 |
| SCFA | Iso valeric acid | 1.69 | 0.430 |
| SCFA | Caproic acid | 96 | <0.001 |

**Table A3.** Pairwise comparisons change in metabolites 6 and 24 months after bariatric surgery

| Type | Variable | Contrast | Ratio | Standaard error | Statistic | p-value |
| --- | --- | --- | --- | --- | --- | --- |
| Inflammation | CRP | 6 months / baseline | 0.275 | 0.0391 | -9.09 | <0.001 |
| Inflammation | CRP | 24 months/baseline | 0.133 | 0.0189 | -14.2 | <0.001 |
| Inflammation | CRP | 24 months / 6 months | 0.484 | 0.0686 | -5.12 | <0.001 |
| Inflammation | SAA | 6 months / baseline | 0.262 | 0.0296 | -11.9 | <0.001 |
| Inflammation | SAA | 24 months/baseline | 0.214 | 0.0242 | -13.7 | <0.001 |
| Inflammation | SAA | 24 months / 6 months | 0.817 | 0.0923 | -1.79 | 0.076 |
| Inflammation | Haptoglobin | 6 months / baseline | 0.758 | 0.0487 | -4.31 | <0.001 |
| Inflammation | Haptoglobin | 24 months/baseline | 0.564 | 0.0362 | -8.91 | <0.001 |
| Inflammation | Haptoglobin | 24 months / 6 months | 0.744 | 0.0478 | -4.6 | <0.001 |
| Inflammation | LPS | 6 months / baseline | 0.804 | 0.0259 | -6.79 | <0.001 |
| Inflammation | LPS | 24 months/baseline | 0.64 | 0.0206 | -13.9 | <0.001 |
| Inflammation | LPS | 24 months / 6 months | 0.796 | 0.0256 | -7.08 | <0.001 |
| Bile acids | CA | 6 months / baseline | 2.32 | 0.573 | 3.4 | <0.001 |
| Bile acids | CA | 24 months/baseline | 1.9 | 0.466 | 2.6 | 0.010 |
| Bile acids | CA | 24 months / 6 months | 0.818 | 0.202 | -0.812 | 0.419 |
| Bile acids | CDC | 6 months / baseline | 2.03 | 0.486 | 2.95 | 0.004 |
| Bile acids | CDC | 24 months/baseline | 1.39 | 0.332 | 1.39 | 0.168 |
| Bile acids | CDC | 24 months / 6 months | 0.687 | 0.165 | -1.57 | 0.120 |
| Bile acids | DCA | 6 months / baseline | 1.19 | 0.2 | 1.01 | 0.314 |
| Bile acids | DCA | 24 months/baseline | 1.16 | 0.195 | 0.902 | 0.369 |
| Bile acids | DCA | 24 months / 6 months | 0.981 | 0.165 | -0.113 | 0.910 |
| Bile acids | GCA | 6 months / baseline | 1.61 | 0.273 | 2.82 | 0.006 |
| Bile acids | GCA | 24 months/baseline | 0.926 | 0.156 | -0.455 | 0.650 |
| Bile acids | GCA | 24 months / 6 months | 0.575 | 0.0974 | -3.27 | 0.001 |
| Bile acids | GCDC | 6 months / baseline | 1.92 | 0.286 | 4.37 | <0.001 |
| Bile acids | GCDC | 24 months/baseline | 1.55 | 0.23 | 2.95 | 0.004 |
| Bile acids | GCDC | 24 months / 6 months | 0.808 | 0.12 | -1.43 | 0.154 |
| Bile acids | GDC | 6 months / baseline | 1.51 | 0.229 | 2.72 | 0.007 |
| Bile acids | GDC | 24 months/baseline | 1.21 | 0.182 | 1.24 | 0.216 |
| Bile acids | GDC | 24 months / 6 months | 0.798 | 0.121 | -1.49 | 0.139 |
| Bile acids | GLC | 6 months / baseline | 0.939 | 0.154 | -0.387 | 0.700 |
| Bile acids | GLC | 24 months/baseline | 1.15 | 0.188 | 0.882 | 0.380 |
| Bile acids | GLC | 24 months / 6 months | 1.23 | 0.202 | 1.26 | 0.209 |
| Bile acids | GLC-3S | 6 months / baseline | 1.31 | 0.175 | 1.98 | 0.050 |
| Bile acids | GLC-3S | 24 months/baseline | 1.78 | 0.239 | 4.33 | <0.001 |
| Bile acids | GLC-3S | 24 months / 6 months | 1.37 | 0.184 | 2.32 | 0.022 |
| Bile acids | TCA | 6 months / baseline | 3.21 | 0.561 | 6.66 | <0.001 |
| Bile acids | TCA | 24 months/baseline | 2.08 | 0.363 | 4.2 | <0.001 |
| Bile acids | TCA | 24 months / 6 months | 0.649 | 0.114 | -2.47 | 0.015 |
| Bile acids | TCDC | 6 months / baseline | 1.65 | 0.3 | 2.76 | 0.007 |
| Bile acids | TCDC | 24 months/baseline | 1.54 | 0.278 | 2.37 | 0.019 |
| Bile acids | TCDC | 24 months / 6 months | 0.931 | 0.169 | -0.395 | 0.694 |
| Bile acids | TDC | 6 months / baseline | 1.31 | 0.245 | 1.43 | 0.156 |
| Bile acids | TDC | 24 months/baseline | 1.09 | 0.204 | 0.462 | 0.645 |
| Bile acids | TDC | 24 months / 6 months | 0.834 | 0.156 | -0.966 | 0.336 |
| Bile acids | UDC | 6 months / baseline | 1.24 | 0.22 | 1.19 | 0.236 |
| Bile acids | UDC | 24 months/baseline | 0.918 | 0.162 | -0.486 | 0.628 |
| Bile acids | UDC | 24 months / 6 months | 0.743 | 0.132 | -1.68 | 0.096 |
| SCFA | Acetic acid | 6 months / baseline | 1.33 | 0.0786 | 4.86 | <0.001 |
| SCFA | Acetic acid | 24 months/baseline | 1.66 | 0.0975 | 8.62 | <0.001 |
| SCFA | Acetic acid | 24 months / 6 months | 1.25 | 0.0735 | 3.72 | <0.001 |
| SCFA | Propionic acid | 6 months / baseline | 0.669 | 0.0749 | -3.59 | <0.001 |
| SCFA | Propionic acid | 24 months/baseline | 1.26 | 0.14 | 2.08 | 0.040 |
| SCFA | Propionic acid | 24 months / 6 months | 1.88 | 0.211 | 5.66 | <0.001 |
| SCFA | Butyric acid | 6 months / baseline | 1.11 | 0.0858 | 1.3 | 0.197 |
| SCFA | Butyric acid | 24 months/baseline | 1.25 | 0.0968 | 2.94 | 0.004 |
| SCFA | Butyric acid | 24 months / 6 months | 1.13 | 0.088 | 1.63 | 0.106 |
| SCFA | Iso-butyric acid | 6 months / baseline | 0.99 | 0.0495 | -0.2 | 0.842 |
| SCFA | Iso-butyric acid | 24 months/baseline | 1.08 | 0.0535 | 1.46 | 0.147 |
| SCFA | Iso-butyric acid | 24 months / 6 months | 1.09 | 0.0543 | 1.65 | 0.101 |
| SCFA | Methyl butyric acid | 6 months / baseline | 0.954 | 0.0432 | -1.03 | 0.305 |
| SCFA | Methyl butyric acid | 24 months/baseline | 0.985 | 0.0443 | -0.342 | 0.733 |
| SCFA | Methyl butyric acid | 24 months / 6 months | 1.03 | 0.0467 | 0.69 | 0.491 |
| SCFA | Iso valeric acid | 6 months / baseline | 1.06 | 0.0828 | 0.683 | 0.496 |
| SCFA | Iso valeric acid | 24 months/baseline | 1.11 | 0.0864 | 1.3 | 0.197 |
| SCFA | Iso valeric acid | 24 months / 6 months | 1.05 | 0.0823 | 0.61 | 0.543 |
| 0.543SCFA | Caproic acid | 6 months / baseline | 1.24 | 0.114 | 2.33 | 0.021 |
| SCFA | Caproic acid | 24 months/baseline | 2.38 | 0.218 | 9.42 | <0.001 |
| SCFA | Caproic acid | 24 months / 6 months | 1.92 | 0.177 | 7.04 | <0.001 |

**Table A4.** Correlation between microbiota alpha diversity and gastrointestinal complaints

| **variable** | **contrast** | **ratio** | **SE** | **df** | **null** | **t.ratio** | **p.value** |
| --- | --- | --- | --- | --- | --- | --- | --- |
| IBS Total | None / Mild | 0,93 | 0,047 | 50 | 1 | -1,44 | 0,155 |
| IBS Total | None / Moderate/severe | 0,952 | 0,0492 | 50 | 1 | -0,961 | 0,341 |
| IBS Total | Mild / Moderate/severe | 1,02 | 0,0492 | 50 | 1 | 0,482 | 0,632 |

**Table A5.** Correlation between metabolites and gastrointestinal complaints

| **variable** | **item** | **type** | **timepoint** | **contrast** | **ratio** | **SE** | **p.value** |
| --- | --- | --- | --- | --- | --- | --- | --- |
| CRP | IBS Total | inflammation | 24 Months | None / Mild | 1,3 | 0,403 | 0,406 |
| CRP | IBS Total | inflammation | 24 Months | None / Moderate/severe | 1,27 | 0,415 | 0,459 |
| CRP | IBS Total | inflammation | 24 Months | Mild / Moderate/severe | 0,983 | 0,292 | 0,954 |
| SAA | IBS Total | inflammation | 24 Months | None / Mild | 1,16 | 0,286 | 0,546 |
| SAA | IBS Total | inflammation | 24 Months | None / Moderate/severe | 1,29 | 0,332 | 0,33 |
| SAA | IBS Total | inflammation | 24 Months | Mild / Moderate/severe | 1,11 | 0,261 | 0,662 |
| Haptoglobin | IBS Total | inflammation | 24 Months | None / Mild | 0,955 | 0,198 | 0,824 |
| Haptoglobin | IBS Total | inflammation | 24 Months | None / Moderate/severe | 0,872 | 0,189 | 0,529 |
| Haptoglobin | IBS Total | inflammation | 24 Months | Mild / Moderate/severe | 0,913 | 0,181 | 0,647 |
| LPS | IBS Total | inflammation | 24 Months | None / Mild | 1,38 | 0,382 | 0,247 |
| LPS | IBS Total | inflammation | 24 Months | None / Moderate/severe | 1,38 | 0,4 | 0,268 |
| LPS | IBS Total | inflammation | 24 Months | Mild / Moderate/severe | 1 | 0,265 | 0,998 |
| CA | IBS Total | bile_acids | 24 Months | None / Mild | 1,94 | 1,01 | 0,212 |
| CA | IBS Total | bile_acids | 24 Months | None / Moderate/severe | 1,91 | 1,05 | 0,242 |
| CA | IBS Total | bile_acids | 24 Months | Mild / Moderate/severe | 0,988 | 0,495 | 0,98 |
| CDC | IBS Total | bile_acids | 24 Months | None / Mild | 2,14 | 1,06 | 0,128 |
| CDC | IBS Total | bile_acids | 24 Months | None / Moderate/severe | 1,85 | 0,959 | 0,238 |
| CDC | IBS Total | bile_acids | 24 Months | Mild / Moderate/severe | 0,864 | 0,409 | 0,759 |
| DCA | IBS Total | bile_acids | 24 Months | None / Mild | 1,04 | 0,329 | 0,898 |
| DCA | IBS Total | bile_acids | 24 Months | None / Moderate/severe | 0,878 | 0,291 | 0,695 |
| DCA | IBS Total | bile_acids | 24 Months | Mild / Moderate/severe | 0,843 | 0,255 | 0,574 |
| GCA | IBS Total | bile_acids | 24 Months | None / Mild | 0,795 | 0,243 | 0,455 |
| GCA | IBS Total | bile_acids | 24 Months | None / Moderate/severe | 0,827 | 0,265 | 0,557 |
| GCA | IBS Total | bile_acids | 24 Months | Mild / Moderate/severe | 1,04 | 0,305 | 0,891 |
| GCDC | IBS Total | bile_acids | 24 Months | None / Mild | 0,907 | 0,247 | 0,723 |
| GCDC | IBS Total | bile_acids | 24 Months | None / Moderate/severe | 0,865 | 0,247 | 0,613 |
| GCDC | IBS Total | bile_acids | 24 Months | Mild / Moderate/severe | 0,953 | 0,249 | 0,855 |
| GDC | IBS Total | bile_acids | 24 Months | None / Mild | 0,796 | 0,229 | 0,43 |
| GDC | IBS Total | bile_acids | 24 Months | None / Moderate/severe | 0,757 | 0,228 | 0,359 |
| GDC | IBS Total | bile_acids | 24 Months | Mild / Moderate/severe | 0,951 | 0,261 | 0,856 |
| GLC | IBS Total | bile_acids | 24 Months | None / Mild | 0,741 | 0,247 | 0,372 |
| GLC | IBS Total | bile_acids | 24 Months | None / Moderate/severe | 0,562 | 0,196 | 0,104 |
| GLC | IBS Total | bile_acids | 24 Months | Mild / Moderate/severe | 0,758 | 0,242 | 0,389 |
| GLC-3S | IBS Total | bile_acids | 24 Months | None / Mild | 0,468 | 0,139 | 0,0131 |
| GLC-3S | IBS Total | bile_acids | 24 Months | None / Moderate/severe | 0,351 | 0,109 | 0,00133 |
| GLC-3S | IBS Total | bile_acids | 24 Months | Mild / Moderate/severe | 0,75 | 0,213 | 0,316 |
| TCA | IBS Total | bile_acids | 24 Months | None / Mild | 0,673 | 0,24 | 0,271 |
| TCA | IBS Total | bile_acids | 24 Months | None / Moderate/severe | 1,17 | 0,438 | 0,671 |
| TCA | IBS Total | bile_acids | 24 Months | Mild / Moderate/severe | 1,74 | 0,594 | 0,108 |
| TCDC | IBS Total | bile_acids | 24 Months | None / Mild | 1,01 | 0,386 | 0,988 |
| TCDC | IBS Total | bile_acids | 24 Months | None / Moderate/severe | 1,39 | 0,558 | 0,416 |
| TCDC | IBS Total | bile_acids | 24 Months | Mild / Moderate/severe | 1,38 | 0,507 | 0,382 |
| TDC | IBS Total | bile_acids | 24 Months | None / Mild | 0,82 | 0,309 | 0,6 |
| TDC | IBS Total | bile_acids | 24 Months | None / Moderate/severe | 1,1 | 0,435 | 0,806 |
| TDC | IBS Total | bile_acids | 24 Months | Mild / Moderate/severe | 1,34 | 0,485 | 0,415 |
| UDC | IBS Total | bile_acids | 24 Months | None / Mild | 0,917 | 0,265 | 0,766 |
| UDC | IBS Total | bile_acids | 24 Months | None / Moderate/severe | 0,677 | 0,205 | 0,203 |
| UDC | IBS Total | bile_acids | 24 Months | Mild / Moderate/severe | 0,738 | 0,204 | 0,277 |
| Acetic acid | IBS Total | scfa | 24 Months | None / Mild | 1,14 | 0,0863 | 0,0902 |
| Acetic acid | IBS Total | scfa | 24 Months | None / Moderate/severe | 1,02 | 0,0811 | 0,781 |
| Acetic acid | IBS Total | scfa | 24 Months | Mild / Moderate/severe | 0,897 | 0,065 | 0,14 |
| Propionic acid | IBS Total | scfa | 24 Months | None / Mild | 1,13 | 0,138 | 0,34 |
| Propionic acid | IBS Total | scfa | 24 Months | None / Moderate/severe | 1,03 | 0,132 | 0,818 |
| Propionic acid | IBS Total | scfa | 24 Months | Mild / Moderate/severe | 0,915 | 0,107 | 0,454 |
| Butyric acid | IBS Total | scfa | 24 Months | None / Mild | 1,22 | 0,167 | 0,146 |
| Butyric acid | IBS Total | scfa | 24 Months | None / Moderate/severe | 0,954 | 0,137 | 0,744 |
| Butyric acid | IBS Total | scfa | 24 Months | Mild / Moderate/severe | 0,78 | 0,102 | 0,0624 |
| Iso-butyric acid | IBS Total | scfa | 24 Months | None / Mild | 1,14 | 0,106 | 0,176 |
| Iso-butyric acid | IBS Total | scfa | 24 Months | None / Moderate/severe | 0,993 | 0,0972 | 0,939 |
| Iso-butyric acid | IBS Total | scfa | 24 Months | Mild / Moderate/severe | 0,873 | 0,0781 | 0,135 |
| Methyl butyric acid | IBS Total | scfa | 24 Months | None / Mild | 1,15 | 0,108 | 0,127 |
| Methyl butyric acid | IBS Total | scfa | 24 Months | None / Moderate/severe | 1,06 | 0,103 | 0,568 |
| Methyl butyric acid | IBS Total | scfa | 24 Months | Mild / Moderate/severe | 0,916 | 0,0816 | 0,327 |
| Iso valeric acid | IBS Total | scfa | 24 Months | None / Mild | 1,21 | 0,178 | 0,203 |
| Iso valeric acid | IBS Total | scfa | 24 Months | None / Moderate/severe | 1,08 | 0,167 | 0,603 |
| Iso valeric acid | IBS Total | scfa | 24 Months | Mild / Moderate/severe | 0,897 | 0,126 | 0,443 |
| Caproic acid | IBS Total | scfa | 24 Months | None / Mild | 0,899 | 0,113 | 0,401 |
| Caproic acid | IBS Total | scfa | 24 Months | None / Moderate/severe | 0,944 | 0,124 | 0,66 |
| Caproic acid | IBS Total | scfa | 24 Months | Mild / Moderate/severe | 1,05 | 0,126 | 0,69 |

**Table A6.** Spearman correlation between microbiota alpha diversity and metabolites

| **variable** | **cor** | **statistic** | **p** | **method** |
| --- | --- | --- | --- | --- |
| Acetic acid | -0,26 | 31300 | 0,0584 | Spearman |
| Butyric acid | -0,13 | 28000 | 0,356 | Spearman |
| CA | -0,069 | 26500 | 0,622 | Spearman |
| CDC | -0,14 | 28400 | 0,303 | Spearman |
| CRP | -0,1 | 27300 | 0,472 | Spearman |
| Caproic acid | -0,12 | 27800 | 0,382 | Spearman |
| DCA | 0,043 | 23700 | 0,758 | Spearman |
| GCA | -0,074 | 26600 | 0,598 | Spearman |
| GCDC | -0,08 | 26800 | 0,568 | Spearman |
| GDC | 0,14 | 21300 | 0,318 | Spearman |
| GLC | 0,28 | 17900 | 0,0425 | Spearman |
| GLC-3S | 0,14 | 21400 | 0,332 | Spearman |
| Haptoglobin | -0,13 | 28000 | 0,355 | Spearman |
| Iso valeric acid | -0,27 | 31600 | 0,0466 | Spearman |
| Iso-butyric acid | -0,22 | 30100 | 0,121 | Spearman |
| LPS | -0,0038 | 24900 | 0,979 | Spearman |
| Methyl butyric acid | -0,28 | 31700 | 0,0431 | Spearman |
| Propionic acid | -0,21 | 29900 | 0,139 | Spearman |
| SAA | -0,17 | 28900 | 0,233 | Spearman |
| TCA | -0,097 | 27200 | 0,489 | Spearman |
| TCDC | -0,14 | 28200 | 0,331 | Spearman |
| TDC | 0,059 | 23300 | 0,672 | Spearman |
| UDC | 0,15 | 21100 | 0,285 | Spearman |

**Table A7.** Correlation between microbiota beta diversity and metabolites.

| **variable** | **ASV** | **log2FoldChange** | **p-value** |
| --- | --- | --- | --- |
| CRP | ASV27 | -3.61 | 0.00403 |
| GCDC | ASV336 | 3 | 0.0417 |
| GDC | ASV157 | 4.37 | 0.0179 |
| Acetic acid | ASV106 | 5.02 | 0.00589 |
| Acetic acid | ASV157 | 4.38 | 0.0289 |
| Acetic acid | ASV165 | 3.37 | 0.0289 |
| Propionic acid | ASV388 | 3.81 | 0.00794 |
| Butyric acid | ASV144 | -3.57 | 0.0425 |
| Butyric acid | ASV311 | 3.31 | 0.0269 |
| Iso-butyric acid | ASV173 | -3.02 | 0.0341 |
| Iso-butyric acid | ASV388 | 4.03 | 0.00792 |
